# Crystal structure of 4,6-di­amino-2-(methyl­sulfan­yl)pyridine-3-carbo­nitrile

**DOI:** 10.1107/S2056989015003114

**Published:** 2015-02-21

**Authors:** Shaaban K. Mohamed, Kyle S. Knight, Mehmet Akkurt, Bahgat R. M. Hussein, Mustafa R. Albayati

**Affiliations:** a Chemistry and Environmental Division, Manchester Metropolitan University, Manchester M1 5GD, England; b Chemistry Department, Faculty of Science, Mini University, 61519 El-Minia, Egypt; c Department of Chemistry, The University of Tennessee at Chattanooga, Chattanooga, TN 37403, USA; d Department of Physics, Faculty of Sciences, Erciyes University, 38039 Kayseri, Turkey; e Chemistry Department, Faculty of Science, Sohag University, 82524 Sohag, Egypt; f Kirkuk University, College of Science, Department of Chemistry, Kirkuk, Iraq

**Keywords:** crystal structure, 4,6-di­amino-2-(methyl­sulfan­yl)pyridine-3-carbo­nitrile, multifunctional pyridines

## Abstract

The title pyrimidine derivative, C_7_H_8_N_4_S, is essentially planar, with a maximum deviation of 0.029 (2) Å from the mean plane of the non-H atoms. In the crystal, mol­ecules are linked by an inter­molecular bifurcated N—H⋯N hydrogen bond between the cyano N atom and the two amino groups, an N—H⋯N hydrogen bond between the two amino groups and a weak C—H⋯π inter­action, forming a three-dimensional network.

## Related literature   

For the abundance of pyridines in pharmaceuticals and natural products, see: Zhang *et al.* (2010 ▸). For various applications of pyridine-containing compounds, see: Murata *et al.* (2003 ▸). For the use of polyfunctional pyridines in preparing a variety of heterocyclic compounds, see: Al-Haiza *et al.* (2003 ▸). For the synthesis of the title compound, see: Abu-Shanab (1999 ▸). For a similar structure, see: Mohamed *et al.* (2014 ▸).
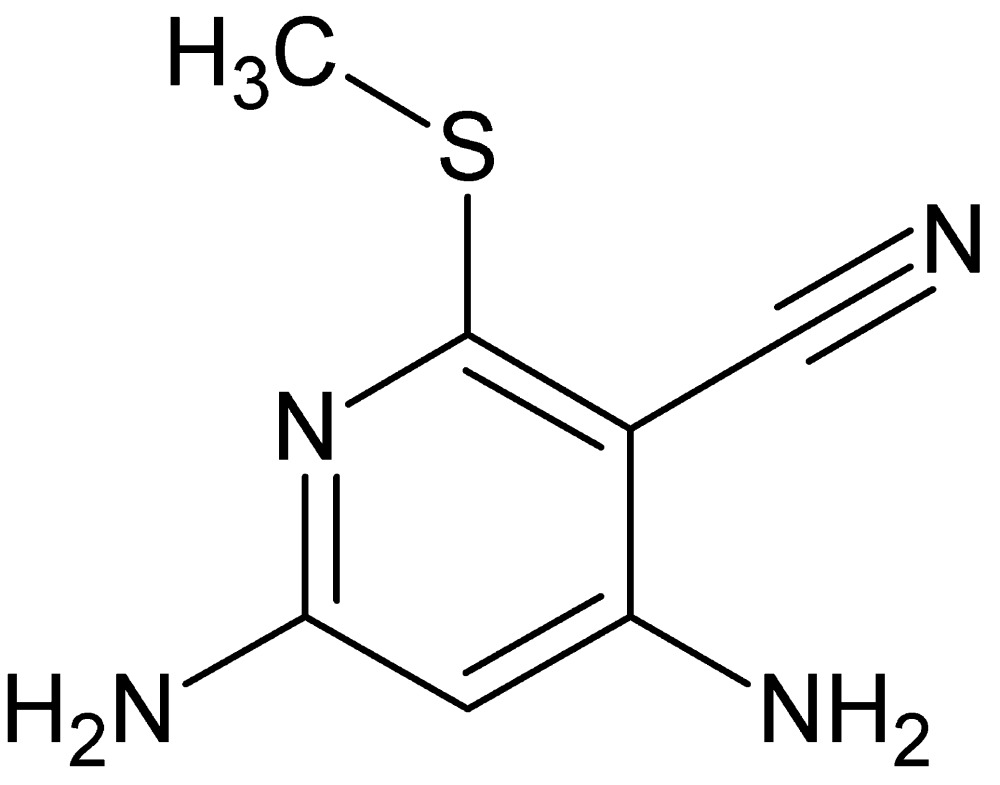


## Experimental   

### Crystal data  

C_7_H_8_N_4_S
*M* *_r_* = 180.23
Orthorhombic, 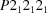

*a* = 5.0863 (7) Å
*b* = 12.698 (2) Å
*c* = 13.069 (2) Å
*V* = 844.1 (2) Å^3^
*Z* = 4
Mo *K*α radiation
μ = 0.33 mm^−1^
*T* = 200 K
0.40 × 0.09 × 0.05 mm

### Data collection  

Bruker SMART X2S benchtop diffractometer
Absorption correction: multi-scan (*SADABS*; Bruker, 2008 ▸) *T*_min_ = 0.833, *T*_max_ = 0.984
9083 measured reflections
1487 independent reflections
1353 reflections with *I* > 2σ(*I*)
*R*_int_ = 0.037

### Refinement  

*R*[*F* ^2^ > 2σ(*F* ^2^)] = 0.026
*wR*(*F* ^2^) = 0.062
*S* = 1.06
1487 reflections
122 parameters
6 restraints
H atoms treated by a mixture of independent and constrained refinement
Δρ_max_ = 0.20 e Å^−3^
Δρ_min_ = −0.13 e Å^−3^
Absolute structure: Flack (1983 ▸)
Absolute structure parameter: 0.01 (4)

### 

Data collection: *APEX2* (Bruker, 2009 ▸); cell refinement: *SAINT* (Bruker, 2009 ▸); data reduction: *SAINT*; program(s) used to solve structure: *SHELXS2014* (Sheldrick, 2008 ▸); program(s) used to refine structure: *SHELXL2014* (Sheldrick, 2008 ▸); molecular graphics: *ORTEP-3 for Windows* (Farrugia, 2012 ▸); software used to prepare material for publication: *PLATON* (Spek, 2009 ▸).

## Supplementary Material

Crystal structure: contains datablock(s) global, I. DOI: 10.1107/S2056989015003114/is5390sup1.cif

Structure factors: contains datablock(s) I. DOI: 10.1107/S2056989015003114/is5390Isup2.hkl

Supporting information file. DOI: 10.1107/S2056989015003114/is5390Isup3.cml

**Fig. 1.**
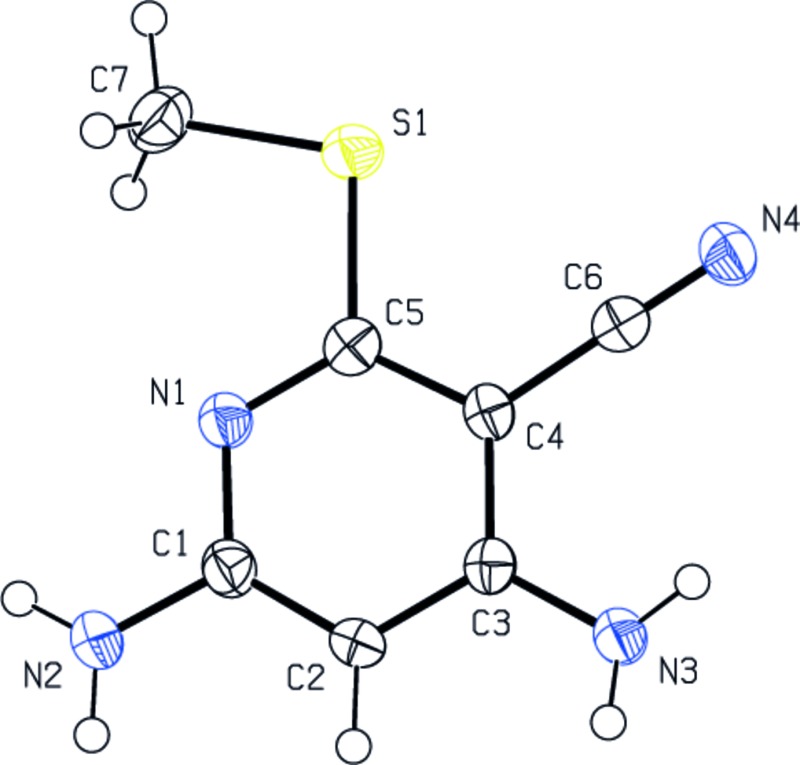
The molecular structure of the title compound with the atom numbering scheme. Displacement ellipsoids for non-H atoms are drawn at the 50% probability level.

**Fig. 2.**
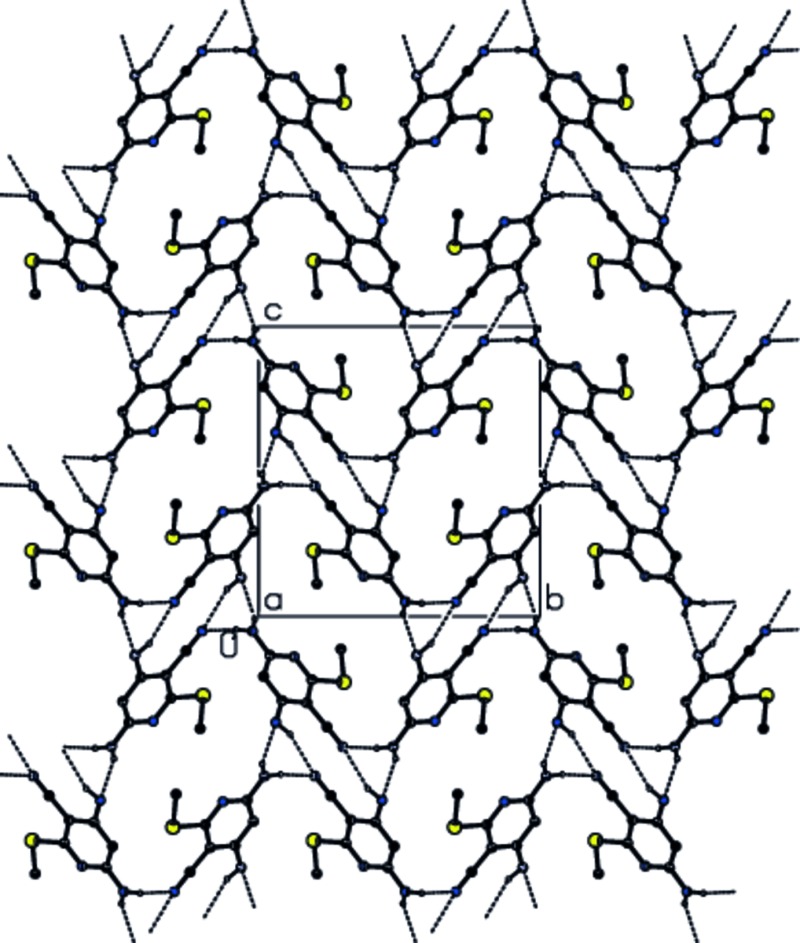
The hydrogen bonding (dashed lines) and packing of the title compound viewed down the a axis.

. DOI: 10.1107/S2056989015003114/is5390fig1.tif
The mol­ecular structure of the title compound with the atom numbering scheme. Displacement ellipsoids for non-H atoms are drawn at the 50% probability level.

a . DOI: 10.1107/S2056989015003114/is5390fig2.tif
The hydrogen bonding (dashed lines) and packing of the title compound viewed down the *a* axis.

CCDC reference: 1049335

Additional supporting information:  crystallographic information; 3D view; checkCIF report

## Figures and Tables

**Table 1:** Hydrogen-bond geometry (, )

*D*H*A*	*D*H	H*A*	*D* *A*	*D*H*A*
N2H2*A*N3^i^	0.86(3)	2.43(3)	3.225(4)	155(3)
N2H2*B*N4^ii^	0.86(2)	2.26(3)	3.083(4)	161(3)
N3H3*B*N4^iii^	0.85(2)	2.31(2)	3.128(3)	161(2)
C7H7*A* *Cg*1^iv^	0.98	2.77	3.552(4)	137

Symmetry codes: (i) 
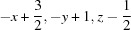
; (ii) 
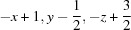
; (iii) 
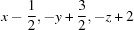
; (iv) 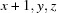.

## supplementary crystallographic information

### S1. Comment

The pyridine ring is a core structure in a number of pharmaceuticals and natural products (Zhang *et al.*, 2010). 2-Amino-3-cyanopyridines have been identified as IKK-β inhibitors (Murata *et al.*, 2003). Besides this, they are important and useful intermediates in preparing variety of heterocyclic compounds (Al-Haiza *et al.*, 2003). Such findings and following to our on-going study on synthesis of bio-active heterocyclic molecules we report in this study the synthesis and crystal structure determination of the title compound.

The molecule of the title compound, Fig. 1, is a tetra-substituted pyrimidine derivative, which is essentially planar with C7–S1–C5–C4, C3–C2–C1–N2, N3–C3–C4–C5 and C6–C4–C3–C2 torsion angles being 180.0 (2), 179.9 (2), 179.0 (2) and 179.7 (2)°, respectively. All bond lengths and bond angles are normal and comparable to those observed in a similar structure (Mohamed *et al.*, 2014). In the crystal structure, intermolecular N—H···N hydrogen bonds and a weak C—H···π interaction feature in the crystal packing (Table 1, Fig. 2).

### S2. Experimental

The title compound was prepared according to the reported method (Abu-Shanab, 1999). Crystals of the product were obtained in a good yield (77%) and were suitable for X-ray diffraction (*M.p.* 426–428 K).

### S3. Refinement

H-atoms attached to carbon were placed in calculated positions (C—H = 0.95–0.98 Å) and refined as riding with *U*_iso_(H) = 1.2 or 1.5*U*_eq_(C) . The H atoms attached to N2 and N3 were found in a difference Fourier map and their positions were refined with bond length and angle restraints of N—H = 0.86 (1) and H···H = 1.40 (3) Å, and with *U*_iso_(H) = 1.5*U*_eq_(N).

### Crystal data

**Table:**

C_7_H_8_N_4_S	*F*(000) = 376
*M**_r_* = 180.23	*D*_x_ = 1.418 Mg m^−^^3^
Orthorhombic, *P*2_1_2_1_2_1_	Mo *K*α radiation, λ = 0.71073 Å
Hall symbol: P 2ac 2ab	Cell parameters from 3713 reflections
*a* = 5.0863 (7) Å	θ = 2.2–25.0°
*b* = 12.698 (2) Å	µ = 0.33 mm^−^^1^
*c* = 13.069 (2) Å	*T* = 200 K
*V* = 844.1 (2) Å^3^	Needle, yellow
*Z* = 4	0.40 × 0.09 × 0.05 mm

### Data collection

**Table:**

Bruker SMART X2S benchtop diffractometer	1487 independent reflections
Radiation source: XOS X-beam microfocus source	1353 reflections with *I* > 2σ(*I*)
Doubly curved silicon crystal monochromator	*R*_int_ = 0.037
ω scans	θ_max_ = 25.0°, θ_min_ = 3.1°
Absorption correction: multi-scan (*SADABS*; Bruker, 2008)	*h* = −6→6
*T*_min_ = 0.833, *T*_max_ = 0.984	*k* = −15→12
9083 measured reflections	*l* = −15→15

### Refinement

**Table:**

Refinement on *F*^2^	Hydrogen site location: mixed
Least-squares matrix: full	H atoms treated by a mixture of independent and constrained refinement
*R*[*F*^2^ > 2σ(*F*^2^)] = 0.026	*w* = 1/[σ^2^(*F*_o_^2^) + (0.0294*P*)^2^ + 0.1689*P*] where *P* = (*F*_o_^2^ + 2*F*_c_^2^)/3
*wR*(*F*^2^) = 0.062	(Δ/σ)_max_ < 0.001
*S* = 1.06	Δρ_max_ = 0.20 e Å^−^^3^
1487 reflections	Δρ_min_ = −0.13 e Å^−^^3^
122 parameters	Absolute structure: Flack (1983)
6 restraints	Absolute structure parameter: 0.01 (4)

### Special details

**Table:**

Geometry. Bond distances, angles *etc*. have been calculated using the rounded fractional coordinates. All su's are estimated from the variances of the (full) variance-covariance matrix. The cell e.s.d.'s are taken into account in the estimation of distances, angles and torsion angles
Refinement. Refinement on *F*^2^ for ALL reflections except those flagged by the user for potential systematic errors. Weighted *R*-factors *wR* and all goodnesses of fit *S* are based on *F*^2^, conventional *R*-factors *R* are based on *F*, with *F* set to zero for negative *F*^2^. The observed criterion of *F*^2^ > σ(*F*^2^) is used only for calculating -*R*-factor-obs *etc*. and is not relevant to the choice of reflections for refinement. *R*-factors based on *F*^2^ are statistically about twice as large as those based on *F*, and *R*-factors based on ALL data will be even larger.

### Fractional atomic coordinates and isotropic or equivalent isotropic displacement parameters (Å^2^)

**Table:**

	*x*	*y*	*z*	*U*_iso_*/*U*_eq_	
S1	1.05518 (13)	0.80355 (6)	0.72687 (5)	0.0304 (2)	
N1	0.8883 (4)	0.62615 (18)	0.63890 (15)	0.0256 (7)	
N2	0.7653 (6)	0.4785 (2)	0.55086 (19)	0.0410 (9)	
N3	0.3276 (4)	0.5616 (2)	0.86570 (18)	0.0282 (7)	
N4	0.6450 (4)	0.7998 (2)	0.94969 (16)	0.0322 (7)	
C1	0.7297 (5)	0.5407 (2)	0.6340 (2)	0.0277 (8)	
C2	0.5369 (5)	0.5181 (2)	0.70666 (18)	0.0268 (8)	
C3	0.5066 (4)	0.5834 (2)	0.79017 (17)	0.0222 (8)	
C4	0.6745 (5)	0.67178 (19)	0.79768 (17)	0.0217 (8)	
C5	0.8576 (4)	0.6892 (2)	0.71847 (18)	0.0238 (7)	
C6	0.6581 (5)	0.7431 (2)	0.88141 (19)	0.0242 (8)	
C7	1.2520 (6)	0.7927 (3)	0.6128 (2)	0.0387 (10)	
H2	0.42720	0.45820	0.69870	0.0320*	
H2A	0.894 (5)	0.487 (3)	0.509 (2)	0.0620*	
H2B	0.682 (6)	0.4202 (18)	0.545 (3)	0.0620*	
H3A	0.210 (5)	0.5167 (19)	0.849 (2)	0.0420*	
H3B	0.286 (6)	0.6117 (17)	0.9060 (19)	0.0420*	
H7A	1.32830	0.72190	0.60900	0.0580*	
H7B	1.39330	0.84510	0.61490	0.0580*	
H7C	1.14140	0.80510	0.55260	0.0580*	

### Atomic displacement parameters (Å^2^)

**Table:**

	*U*^11^	*U*^22^	*U*^33^	*U*^12^	*U*^13^	*U*^23^
S1	0.0274 (3)	0.0303 (4)	0.0334 (3)	−0.0054 (3)	0.0014 (3)	−0.0023 (3)
N1	0.0259 (13)	0.0261 (12)	0.0249 (11)	0.0002 (10)	0.0014 (9)	−0.0017 (10)
N2	0.0562 (18)	0.0331 (16)	0.0338 (14)	−0.0117 (13)	0.0185 (12)	−0.0121 (12)
N3	0.0290 (13)	0.0291 (13)	0.0266 (12)	−0.0026 (11)	0.0033 (10)	−0.0050 (10)
N4	0.0355 (12)	0.0340 (14)	0.0270 (11)	−0.0016 (12)	−0.0010 (10)	−0.0054 (12)
C1	0.0328 (15)	0.0254 (15)	0.0248 (13)	0.0026 (12)	0.0010 (11)	−0.0012 (12)
C2	0.0305 (14)	0.0224 (14)	0.0275 (13)	−0.0049 (12)	0.0014 (12)	−0.0042 (11)
C3	0.0222 (15)	0.0245 (14)	0.0200 (12)	0.0035 (10)	−0.0029 (10)	0.0026 (11)
C4	0.0213 (12)	0.0242 (15)	0.0195 (12)	0.0044 (10)	−0.0036 (9)	−0.0002 (10)
C5	0.0207 (12)	0.0268 (13)	0.0240 (11)	0.0031 (11)	−0.0051 (10)	0.0041 (13)
C6	0.0207 (12)	0.0261 (14)	0.0258 (14)	0.0003 (11)	−0.0032 (11)	0.0034 (12)
C7	0.0364 (15)	0.045 (2)	0.0347 (15)	−0.0091 (15)	0.0057 (12)	0.0038 (15)

### Geometric parameters (Å, º)

**Table:**

S1—C5	1.769 (3)	N2—H2B	0.86 (2)
S1—C7	1.801 (3)	N3—H3A	0.86 (2)
N1—C1	1.354 (3)	C3—C4	1.414 (3)
N1—C5	1.322 (3)	N3—H3B	0.85 (2)
N2—C1	1.355 (4)	C4—C5	1.410 (3)
N3—C3	1.371 (3)	C4—C6	1.423 (3)
N4—C6	1.149 (3)	C2—H2	0.9500
C1—C2	1.395 (4)	C7—H7A	0.9800
C2—C3	1.379 (3)	C7—H7B	0.9800
N2—H2A	0.86 (3)	C7—H7C	0.9800
			
C5—S1—C7	101.63 (14)	C5—C4—C6	120.2 (2)
C1—N1—C5	116.9 (2)	C3—C4—C5	118.2 (2)
N1—C1—N2	115.2 (2)	C3—C4—C6	121.6 (2)
N1—C1—C2	123.5 (2)	N1—C5—C4	124.1 (2)
N2—C1—C2	121.3 (2)	S1—C5—N1	118.61 (17)
C1—C2—C3	119.6 (2)	S1—C5—C4	117.28 (18)
C1—N2—H2A	123 (2)	N4—C6—C4	179.3 (3)
C1—N2—H2B	121 (3)	C1—C2—H2	120.00
H2A—N2—H2B	115 (3)	C3—C2—H2	120.00
N3—C3—C2	121.5 (2)	S1—C7—H7A	109.00
N3—C3—C4	120.8 (2)	S1—C7—H7B	109.00
C2—C3—C4	117.7 (2)	S1—C7—H7C	109.00
C3—N3—H3A	114.6 (18)	H7A—C7—H7B	109.00
C3—N3—H3B	117.3 (18)	H7A—C7—H7C	110.00
H3A—N3—H3B	119 (3)	H7B—C7—H7C	110.00
			
C7—S1—C5—C4	180.0 (2)	C1—C2—C3—N3	−177.1 (2)
C7—S1—C5—N1	−0.5 (2)	N3—C3—C4—C5	179.0 (2)
C1—N1—C5—C4	0.9 (4)	N3—C3—C4—C6	−2.3 (4)
C1—N1—C5—S1	−178.60 (18)	C2—C3—C4—C5	1.5 (3)
C5—N1—C1—C2	1.2 (4)	C2—C3—C4—C6	−179.7 (2)
C5—N1—C1—N2	179.4 (2)	C3—C4—C5—N1	−2.3 (4)
N2—C1—C2—C3	−179.9 (2)	C6—C4—C5—S1	−1.6 (3)
N1—C1—C2—C3	−1.8 (4)	C6—C4—C5—N1	179.0 (2)
C1—C2—C3—C4	0.4 (3)	C3—C4—C5—S1	177.24 (18)

### Hydrogen-bond geometry (Å, º)

**Table:**

*D*—H···*A*	*D*—H	H···*A*	*D*···*A*	*D*—H···*A*
N2—H2*A*···N3^i^	0.86 (3)	2.43 (3)	3.225 (4)	155 (3)
N2—H2*B*···N4^ii^	0.86 (2)	2.26 (3)	3.083 (4)	161 (3)
N3—H3*B*···N4^iii^	0.85 (2)	2.31 (2)	3.128 (3)	161 (2)
C7—H7*A*···*Cg*1^iv^	0.98	2.77	3.552 (4)	137

Symmetry codes: (i) −*x*+3/2, −*y*+1, *z*−1/2; (ii) −*x*+1, *y*−1/2, −*z*+3/2; (iii) *x*−1/2, −*y*+3/2, −*z*+2; (iv) *x*+1, *y*, *z*.
