# Supplementary figures and images for: Crystal structure of 4,6-di­amino-2-(methyl­sulfan­yl)pyridine-3-carbo­nitrile
Source: Acta Crystallogr E Crystallogr Commun. 2015 Feb 21;71(Pt 3):o197–8. doi: 10.1107/S2056989015003114 (PMC4350697; doi:10.1107/S2056989015003114)

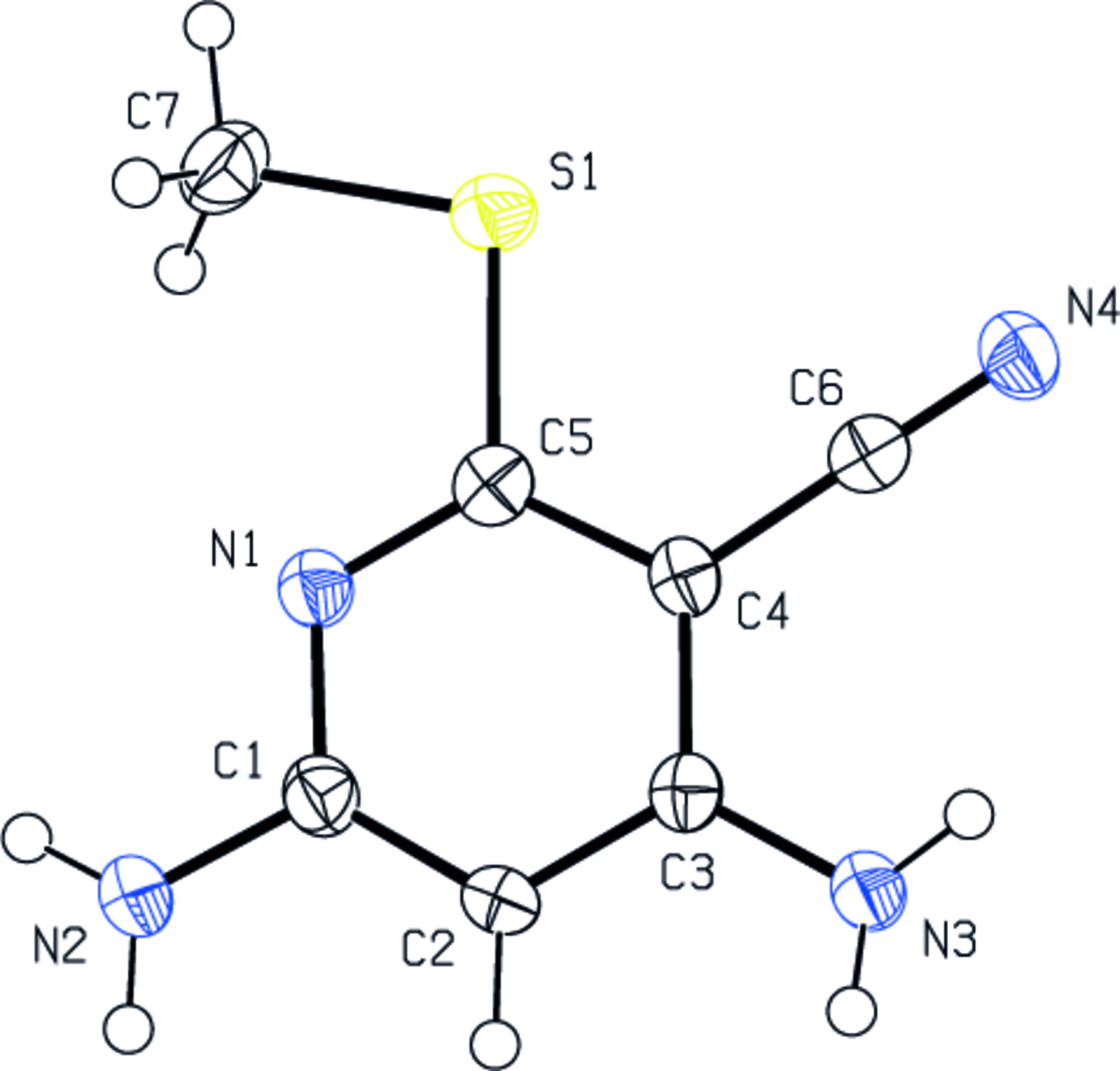

Supplement: Supplementary file 4 [file e-71-0o197-fig1.tif]

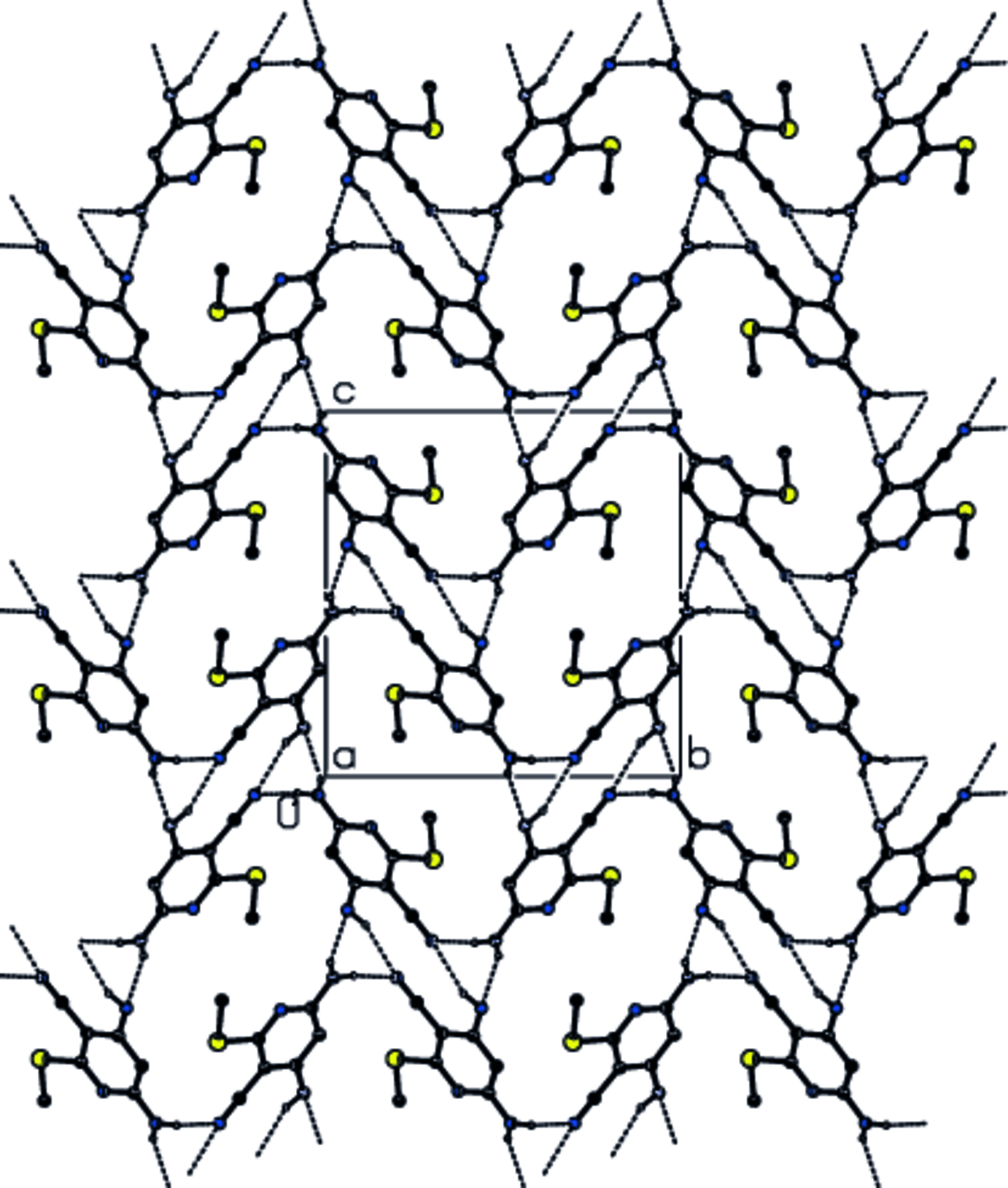

Supplement: Supplementary file 5 [file e-71-0o197-fig2.tif]
